# SAlBi educa (Tailored Nutrition App for Improving Dietary Habits): Initial Evaluation of Usability

**DOI:** 10.3389/fnut.2022.782430

**Published:** 2022-04-19

**Authors:** Marina Gonzalez-Ramirez, Angela Cejudo-Lopez, Mauricio Lozano-Navarrete, Elena Salamero Sánchez-Gabriel, M. Alfonso Torres-Bengoa, Manuel Segura-Balbuena, Maria J. Sanchez-Cordero, Mercedes Barroso-Vazquez, Francisco J. Perez-Barba, Ana M. Troncoso, M. Carmen Garcia-Parrilla, Ana B. Cerezo

**Affiliations:** ^1^Fundación Pública Andaluza para la Gestión de la Investigación en Salud de Sevilla (FISEVI), Hospital Universitario Virgen Macarena, Sevilla, Spain; ^2^Departamento de Nutrición y Bromatología, Toxicología y Medicina Legal, Facultad de Farmacia, Universidad de Sevilla, Sevilla, Spain; ^3^Centro de Salud Bellavista, Distrito Sanitario de Atención Primaria Sevilla, Sevilla, Spain; ^4^UGC Salud Pública Sevilla, Distrito Sanitario de Atención Primaria Sevilla, Área de Promoción de la Salud, Sevilla, Spain; ^5^Centro de Salud Puerta Este “Dr. Pedro Vallina”, Distrito Sanitario de Atención Primaria Sevilla, Sevilla, Spain; ^6^Centro de Salud Esperanza Macarena, Distrito Sanitario de Atención Primaria Sevilla, Sevilla, Spain; ^7^Centro de Salud Los Bermejales, Distrito Sanitario de Atención Primaria Sevilla, Sevilla, Spain

**Keywords:** nutrition education, text messaging, mobile application, healthy diet, Mediterranean diet, feedback, mHealth

## Abstract

In recent years, the use of applications to improve dietary habits has increased. Although numerous nutrition apps are available on the market, only few have been developed by health and nutrition professionals based on scientific evidence and subsequently tested to prove their usability. The main objective of this study was to design, develop, and evaluate the usability of a tailored nutrition application to be used to promote healthy eating habits. In order to decide app design and content, three focus groups took place with fifteen professionals from primary healthcare, nutrition, and food science and computer science, as well as expert users. For the general and feedback message design, a reference model based on the scientific literature was developed. To address the multi-perspective approach of users' and external healthcare professionals' feedback, a one-day pilot testing with potential users and healthcare professionals was conducted with four focus groups. To evaluate the relevance and potential usability of the app a 1-month pilot test was conducted in a real-life environment. A total of 42 volunteers participated in the one-day pilot testing, and 39 potential users participated in the 1-month pilot test. The SAlBi educa app developed includes an online dietary record, a self-monitoring tool to evaluate dietary patterns, general and feedback messages, and examples of traditional Mediterranean recipes. The usability study showed that volunteers think that SAlBi educa is pleasant (59%) and easy to learn to use (94%). Over 84% of the volunteers declared that the nutritional messages were clear and useful. Volunteers stated that general and tailored recommendations, as well as self-monitoring, were SAlBi educa's most motivating and useful features. SAlBi educa is an innovative, user-friendly nutritional education tool with the potential to engage and help individuals to follow dietary habits based on the Mediterranean model.

## Introduction

Overweight and obesity are public health problems worldwide and have a significant impact on non-communicable diseases such as diabetes, hypertension, dyslipidemias, cancer, and cardiovascular diseases (1). The latest World Health Organization (WHO) data reveal that worldwide, 39% of adults are overweight and 13% are obese (2). The United Nations General Assembly High-Level Meeting on Prevention and Control of Non-communicable Diseases (NCDs) recognized the importance of reducing consumption of non-nutritious foods rich in fats, sugars, and salt, and physical inactivity, while increasing consumption of fruits and vegetables, legumes, whole grains, and nuts in order to address the problem of overweight- and obesity-related major non-communicable diseases (3).

The WHO states that the individual responsibilities of a population can only have a full effect when there is access to a healthy lifestyle. Dietary counseling in primary care is a powerful tool since it is the citizens' first point of contact. Its objective is to raise citizens' awareness of healthy eating habits, calorie requirements, calorie intake, and physical activity, as well as impart a correct balance of nutrient intake. Although dietary advice in primary care is one of the main strategies in the fight against overweight and obesity, it presents some limitations, such as accessibility and the time required of health professionals in order to provide nutritional counseling and assessments (4).

The latest WHO report on the global situation of non-communicable diseases, published in 2014 (5), stresses the urgent need for further research to assess the effectiveness of interventions aimed at reducing overweight and preventing obesity. To succeed, this requires a multidisciplinary and innovative approach, making the best use of new technologies (6).

The use of information and communication technologies (ICT) has been proven to have a positive influence on reinforcing and contributing to better health and changes in lifestyle (7). In recent years, the popularity of applications to promote good health and healthy eating habits has increased (8). The aim is to create mobile platforms that act both as a source of critical information and a motivator for users to reinforce and complement nutritional interventions in order to achieve the objectives of a healthy diet (6).

The effectiveness of a food diary, physical activity recorder, and self-evaluation app features for reducing abdominal obesity and the percentage of body fat is relatively modest, but not statistically significant (9, 10). Gamification is a promising methodology for stimulating changes toward healthy dietary habits (11). Only a few studies have been conducted to prove its efficiency. Recently, gamification has been shown to facilitate engagement and refection on nutritional topics enjoyably (12). However, the feedback mechanism has been the most widely tested feature regarding effectiveness. Apps that include feedback messages are more effective in reducing body weight, waist–hip index ratio, and body mass index (BMI) than general nutritional information or the use of standard nutrition apps, lowering BMI by up to 5% in 3 months (13–17). They have also been shown to increase nutritional knowledge, improve dietary quality, reduce portion size, and increase the duration of physical activity. The effectiveness of this system has been attributed to the fact that the population appreciated individualized feedback more positively. Such a system elicits more attention, and its feedback is processed more intensively. Available data suggest the following mechanisms for its rationale: a better exposure to and more intensive cognitive processing of the educational information, as well as greater personal relevance of the message and the self-evaluation properties of tailored feedback (18–21). Petty and Cacioppo's elaboration likelihood model (ELM) (22) had already asserted that health information might be processed *via* central or peripheral routes. The central route requires more cognitive processing, leads more readily to lasting attitude changes, and is stronger when personal information is taken into consideration (18). In fact, some authors suggest that tailored feedback is more likely to be processed centrally, leading to more positive, more personally relevant, more strongly motivational thoughts and more reflections on self-assessment—all of which possess the potential to change health-related behavior patterns (21). In addition, tailored feedback *via* mobile phones has the advantage that it can be applied to a large group of people at a relatively low cost (18). However, the precise features of the messages (length, frequency, timing, etc.) have yet to be agreed upon.

A larger number of apps might increase screen time, which is itself associated with overweight, obesity, and physical inactivity (23). However, Delli Bovi et al. (24) recently showed that when a healthy lifestyle application was used, there was no significant difference in screen time in the control group and the test group.

Although numerous nutrition apps are available for downloading onto users' smartphones, few have been based on scientific evidence, developed by health and nutrition professionals, subsequently tested to prove their usability and efficiency in terms of improving healthy eating habits, and referenced in the literature (6, 25–27). A number of these applications focus exclusively on increasing or decreasing the consumption of a food group, such as fruits and vegetables or sugary drinks and juices (27–30). A few apps do in fact include a daily food record, nutritional evaluations, and feedback (6, 26, 31, 32). Yet, as far as we know, none of them include sugar intake evaluation, caloric distribution throughout the day, or suggest examples of dishes or foods to balance the user's diet. However, to prevent overweight and obesity, the WHO and the European Food and Safety Authority (EFSA) have recently recommended that sugar intake should not exceed 10% of total energy intake (33, 34). Caloric distribution throughout the day is also crucial since greater eating frequency has been associated with a significantly lower risk of obesity in several cross-sectional studies within free-living populations. It also seems to be related to an improved risk status for non-communicable diseases such as diabetes (35–37). In a cross-sectional analysis within the prospective Seasonal Variation of Blood Cholesterol Study (SEASONS) in Worcester County (Massachusetts; *n* = 499; 50.3% men; mean age, 48 years), individuals who ate ≥4 times a day had a significantly lower risk of obesity (OR, 0.55; 95% CI, 0.33–0.91) compared with individuals who ate ≤3 times a day (35). Additionally, more frequent eating (≥4 times a day) was not associated with risk of diabetes mellitus independently of BMI, compared with men who ate three meals a day (36). Therefore, greater eating frequency seems to be related to improved risk status (37). There is also a consensus in terms of fiber intake (>25 g/day) to reduce the risk of NCDs (38). This evidence underpins incorporating their monitoring into an app.

The Mediterranean diet is currently the reference for a healthy diet model. It is characterized by the use of virgin olive oil as the main fat and an abundance of vegetable foods, legumes, fruits, and nuts, as well as a high level of fish and seafood consumption (39). In fact, UNESCO declared it an Intangible Cultural Heritage in 2010 (40). Although, to the best of our knowledge, the benefits of the Mediterranean diet have been widely demonstrated (41, 42), no nutrition app reviewed in the literature includes and recommends recipes and dishes typical of the Mediterranean diet.

The objective of this study was to design, develop, and test the feasibility of an innovative, tailored nutrition app as a complementary tool for the nutritional advice given in primary healthcare based on the Mediterranean diet. We have developed a dietary self-monitoring nutrition application called SAlBi educa, named based on the Spanish terms for health, diet, wellness, and education (Salud, Alimentación, Bienestar, and educación). It includes i) a daily food recorder, ii) a database with more than 300 recipes typical of the Mediterranean diet, incorporating their nutritional information, iii) daily and weekly nutrient and energy evaluation (proteins, fats, carbohydrates, sugar, fiber, and caloric distribution throughout the day), and iv) innovative tools such as notifications and tailored feedback messages concerning the user's diet with recipe suggestions to improve it.

## Materials and Methods

### Design and Development of the SAlBi educa App

The design and development process of the SAlBi educa App followed four main stages: i) collecting information and requirements from healthcare and non-healthcare professionals on nutrition education practices; ii) prototyping the app features based on the selected requirements and a review of the nutrition education apps already available in the literature; and iii) deploying the testing and usability intervention in a formative evaluation study among target volunteers for further app refinement.

SAlBi educa's content and design were discussed in three 90-min focus groups with fifteen participants, comprising eight primary healthcare professionals (family doctors, nurses, and public health and health promotion technicians), three professors and lectures in the nutrition and food science fields, two expert users (pharmacy undergraduates), and two telecommunication engineers. An expert with extensive experience moderated the focus groups. The discussion in the first focus group concentrated on tools to be included in the app and its interface style. The second focus group concentrated on the parameters to be included in the personalized evaluation tool, while the third group focused on the type of message, its characteristics, and its delivery. The agreed suggestions and preferences were taken down by an assistant notetaker during the focus groups and then subsequently implemented in the app.

The mathematical algorithms included in SAlBi educa are based on dietary recommendations from the WHO and the EFSA (Table 1). Additionally, the Spanish Food Composition Database (BEDCA) (46) and the nutritional information on food labeling were used. The Mediterranean diet decalogue was followed for incorporating the Mediterranean meals (47). These recipes were abundant in vegetables, legumes, cereals, and fish, and were always prepared using olive oil. The lean meat was included in small portions as part of a vegetable and cereal-based meal. To establish the number of meals per day, we have followed national and international guidelines. National public *Food-Based Dietary Guidelines* for adults in the EU, Iceland, Norway, Switzerland, and the United Kingdom gathered recommendations on diet and lifestyle habits. Many countries (Bulgaria, Croatia, Czechia, Hungary, Malta, Poland, Slovakia, Slovenia, Spain, and the UK) include information on the number of meals per day, recommending the food to be distributed among 3–5 meals: three main daily meals (breakfast, lunch, and dinner) and two small meals (morning and afternoon snacks) (48). Additionally, the Dietary Guidelines for Americans, 2020–2025 recommend an eating plan that helps manage body weight with up to 6 meals (breakfast, snack, lunch, snack, dinner, and snack) (49). These public healthy eating recommendations are based on scientific evidence (35–37). SAlBi educa was developed to support Android and iOS and was set up in Spanish, although the screenshots shown in this study were translated for purposes of clarity.

**Table 1:** Recommendations used for the evaluation tool algorithms.

**Variables**	**Amount/Percentage of energy intake**	**References**
Number of meals	5 per day	(38)
Calories intake distribution	25% Breakfas	(38)
	10% Midmorning snack	
	30% Lunch	
	10% Afternoon snack	
	25% Dinner	
Fats	25–35%	(43)
Carbohydrates	45–60%	(34)
Sugars	<10%	(33)
Proteins	10–15%	(44)
Fiber	25 g	(34)
Fruit and vegetables	5 per day	(45)

### Message Development

Among the unique tools included in SAlBi educa are both the general and tailored feedback text messages. Based on scientific evidence, a model for developing and designing the messages was established. First, a review of the literature using several databases, such as PubMed, Web of Science, and Scopus, was conducted. This search occurred between 30th January and 10th April 2019 and used the following keywords: “app,” “nutrition,” “feedback,” and “text messages.” A total of 35 results were found in the Web of Science, 36 in Scopus, and 66 in PubMed databases. The inclusion criteria were that the studies in question did in fact prove an improvement in dietary habits while showing users' preferences as well as message features, such as length, content, tone, register, timing, and frequency. A total of 15 studies were thus selected for the review (Table 2). When incorporating text messages into the app, the main aspects were as follows: time, frequency, number, length, age, target group, individualization, customization and personalization, tone, and content.

**Table 2:** Features and main results of general and feedback messages included in different studies.

**App name**	**Objective**	**Features**	**Main results**	**References**
My fitness pal	Weight management	General message topics: Nutritional knowledge, good and not so good food choices, and predictions of future body weight based on daily intake	Although most users rated the messages positively, some expressed concern regarding: i) obvious statements, ii) indication of poor food choices despite users' low consumption of the same, iii) long-term weight predictions based on current daily intake and iv) negative messages.	(8)
Children eating well (CHIEW)	To increase the intake of fruit, vegetables, fiber, and water and reduce the intake of sugary drinks, through parental' information.	- General message topics: Healthy snack recipes; beverage recommendations (including milk); child appropriate snacks; introduction of new foods; and reminders to use the application -Message frequency: Varied number of notifications weekly. Each message was repeated twice every 3 months. - Time of the messages: 10:00 am, although the user could changed it	- New fruits and vegetables introduced - Sugary drinks reduced and substituted by water - Parents offered their children more fruit and vegetables - Improvement in diet	(29)
ONCOFOOD	To improve the dietary goals of cancer patients.	-Daily diet record reminder messages (9:00, 13:00 and 19:00) -Weight record and weekly appetite parameter reminder message by acoustic signal at 17:00.	Those who used the application met 100% of the protein, fat, and energy requirements, compared to the control group that did not reach this goal. Compared to the control group that lost weight (1.03 kg vs. −1.46 kg) as well as muscle mass (0.58 kg vs. −0.61 kg) the group that used the application gained weight significantly	(50)
PYNC	To promote healthy habits (physical activity, healthy weight, calorie intake reduction, healthy diet) for reducing the risk of breast cancer	-General message topics: educational information on the benefits of weight loss through healthy eating, increasing consumption of fruit and vegetables, reducing intake of sugar or red meat, limiting alcohol intake, promoting physical activity, etc.) -Feedback message about calories burnt -Daily intake reminder messages and challenges reminders (every hour g if you had not reached the 250-step challenge)	Volunteers preferred simple language, visually presented information, easy-to-prepare healthy recipes, suggestions for grocery shopping or refrigerator ordering, and strategies for interpreting food labeling	(26)
Txt4happy kids	To increase fruit and vegetables consumption in families with children	General messages: -Frequency: two messages / week -Length: 160 characters -About benefits of consuming fruit and vegetables -Strategies to promote the consumption of fruit and vegetables (e.g., making purchases, cooking together, leading by example, etc.) -Healthy recipes -Fruit and vegetable price reduction advertisements	92% of parents offered more fruit and vegetables to their children because they were aware of the benefits. 86% tried to follow a healthy diet. 85% tried different recipes with fruit and vegetables. 81% were concerned about what their children were consuming. Moreover, 83% agreed that the presence of fruit and vegetables at home had increased since the study. Although, message strategy alone does not seem enough to increase fruit and vegetable consumption	(30).
Body quest parent	To prevent obesity and improve healthy eating habits in children (increased vegetable consumption)	General messages: -Frequency: three messages/week -Content aimed at changing behavior patterns regarding vegetable consumption -Preparing vegetable dishes at home	Significant increase in vegetables consumption	(25)
	To decrease consumption of sugary drinks and juices.	-Messages with recommendations, motivation, and advice on progress (3–4/ week) -Weekly feedback messages about consumption	Significant decrease of 287 mL/day compared to the control group (50 mL/day) and weight decrease by 2.4 kg compared to a gain of 0.9 kg in the control group	(28)
	To increase knowledge on Type 2 Diabetes: prevention, treatment, and management	General messages: -Frequency: two times/week, every 3 months -Length: 160 characters -Content: information on type 2 diabetes	Significant increase in knowledge about the pathology among the intervened group compared to the control group.	(51)
	To promote nutritional behavioral changes in children through parents.	General messages: -Information to promote reflection, discussion, and action -Content: specific portions of healthy foods for different ages, healthy recipes, specific information on nutritional advice and education on healthy eating for children -Includes links for more information: eating habits, dietary quality, preparation of dishes and recipes, family menus and healthy snacks Length: 160 characters	Both parents, experts, and their group evaluated the clarity, usability, and relevance of the messages with 4 points out of 5	(52)
	To increase fruit and vegetables consumption. To reduce the consumption of food with a low nutrient density and sugary drinks	Feedback messages: -Frequency: 1 message/week -Features: personalized with the name of each user, motivational, suggestion of healthy alternatives, including nutritional advice and simple concepts General messages: -Frequency: 1–2 times/week -Between 4 p.m. and 6 p.m. -Features: examples of everyday situations of higher risk of consuming unhealthy foods and healthy suggestions to avoid it, recipe links or nutritional information	Significant reduction in weight (1.7 kg) in experimental group; consumption of low nutrient density foods in experimental group (1.4 servings/day, in men) and consumption of sugary drinks (0.2 servings/day) in experimental group regarding control, in women	(14)
TreC-LifeStyle	To provide nutritional education for families with overweight children	-Daily reports summarizing the deviations and adequacy of their dietary intake with respect to a healthy diet -General messages with nutritional content: advice on a healthy diet, with a frequency of one message/day	The results showed a good adherence to the app with> 90% of the meals recorded over 6 weeks, demonstrating a good acceptance app over the 6 weeks. The parents declared usability as very positive Participants stated that they were influenced by the feedback provided	(6)
	To reduce the weight of an obese population	-Feedback messages: After recording weight, the application automatically provided users with a personalized feedback message about their progress and tips	Significant weight loss in the group intervened: 43% of the patients in the intervention group were more likely to lose 5% or more of their original weight at 6 months, compared to 6% of the patients in the usual care control group	(53)
	To study the tone, content, and length of nutritional messages	General messages: -Content: recommendations on fruit and vegetables, fast food, and alcohol -Length: 2-3 sentences -Tone: empathetic, authoritarian, colloquial, based on solutions or healthy alternatives	22 and 29% of respondents indicated that the empathetic tone and the messages based on solutions or healthy alternatives, respectively, were the most persuasive strategy to produce improvements in diet, compared to the others that obtained a lower percentage of choice.	(54)
Diet-A	To monitor intake in adolescents	-Registration reminders (11:00, 15:00, and 20:00) -Feedback message topics: energy, carbohydrates, protein, fat, saturated fat, calcium and sodium	61.9% of users said they were satisfied after using the application for monitoring their intake Significant reduction in sodium intake	(31)
	To increase vegetable consumption among young adults.	General messages: 1–2 sentences; tips on how to introduce vegetables in the weekly menu; motivational message describing the healthy benefits of fruit	The text messages promoted motivational changes in the consumption of fruit and vegetables	(55)

The nutritional information available in the Healthy Eating Guide for Primary Care and Citizen Groups (56) and the Spanish Food Composition Database (BEDCA) (46) were used for text message development. The contents of the feedback messages, designed *ex profeso* for SAlbi educa, were as follows: i) fruits and vegetables, sugar, carbohydrates, proteins, fiber, and fats intake, suggesting alternate products where appropriate; ii) caloric distribution throughout the day, suggesting alternatives on how to balance it; and iii) congratulating and encouraging users when they follow the recommendations. The messages have been designed for cases when the user's intake is within, above, or below the healthy range. The messages were designed to have an encouraging tone when there had been a satisfactory result for each nutritional parameter, or to be motivational in cases where these parameters needed to be improved. In all cases, the messages were personalized with the user's name. The messages were descriptive and evaluative, and their communicative style was designed to encourage autonomy and empower users (14, 50, 54).

General messages include ones on nutritional knowledge (e.g., the main sources of nutrients for the different food groups included), recipes for certain food groups (vegetables, fruits, legumes, cereals, etc.), and on physical activity, as well as focusing on promoting more direct behavioral changes.

### Users' Feedback and Usability of the App

During the development phase, SAlBi educa was subjected to proofs of concept using data science tools for monitoring, storage, and analysis, such as Google Firebase and BigQuery. Additionally, the app was subjected to a pilot test to address the multi-perspective approach of users' and external healthcare professionals' feedback. A total of four focus groups were organized in four different primary healthcare centers in Sevilla. Participants were recruited among the patients (app target population) and the professionals of the four abovementioned primary healthcare centers in June 2019.

**Figure 1:**
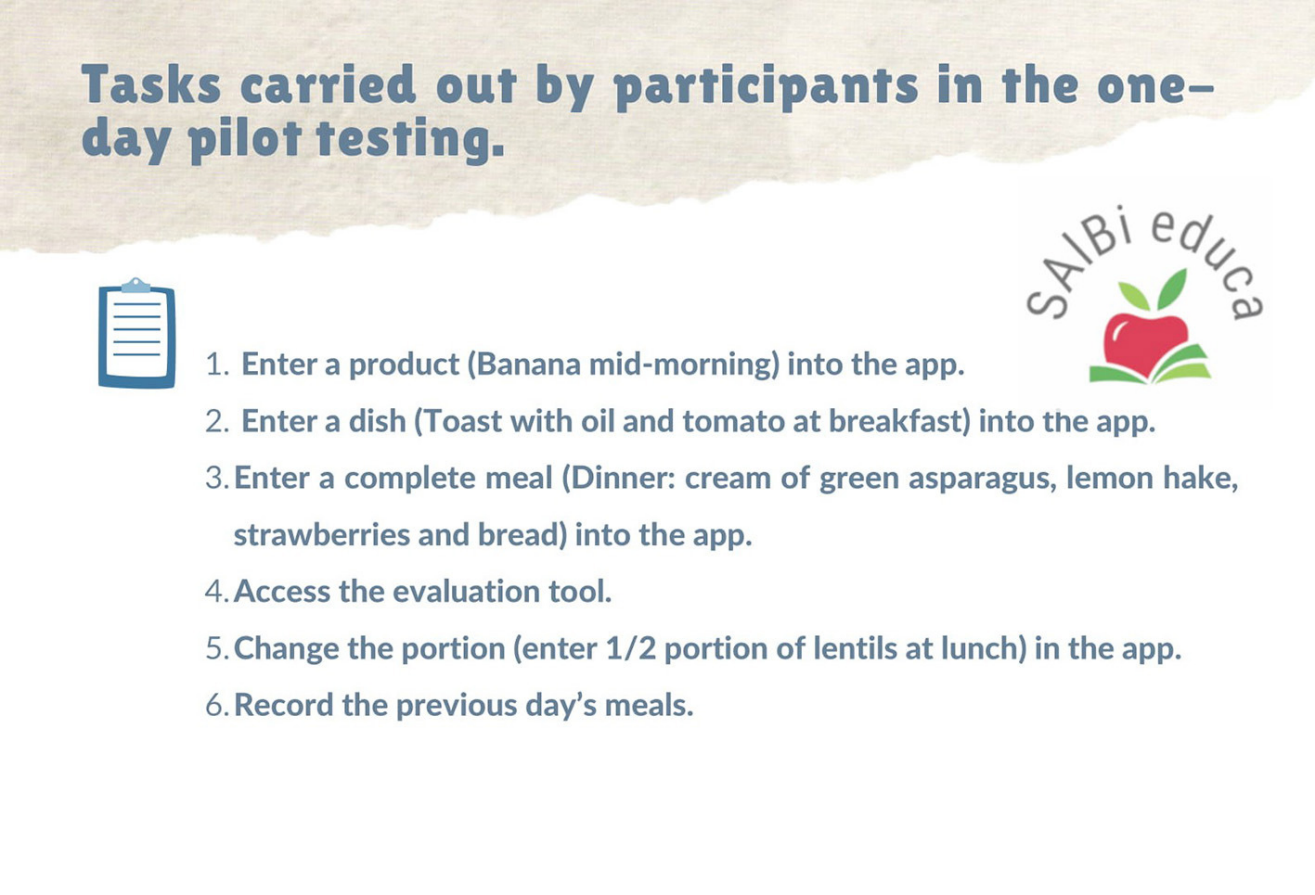
Tasks carried out by participants in the one-day pilot testing.

The specific exclusion criteria were as follows: a) people whose physical or mental state made it impossible for them to complete the questionnaires and use the application; b) speakers of a language other than Spanish that made it impossible for them to use the application and understand the questionnaires correctly; c) people aged under 18 years; and d) people who do not have a smartphone on which to install and use the application. Individuals meeting any of these exclusion criteria were not selected. A total of 42 participants were recruited to conduct a 1-day pilot test. They were invited to take part in a focus group to test a nutritional application. An expert with extensive experience moderated the focus groups. All participants first received written information about the objectives of both the session and the SAlBi educa app. Instructions on how to use the app were not provided. Secondly, the participants were assigned six tasks to be completed through the app (Figure 1). Once they had finished the tasks, they automatically received corresponding tailored feedback messages, as well as general ones. Finally, participants completed a self-administered sociodemographic (including age, weight, height, educational level, or gender) and a usability questionnaire to test user satisfaction and to obtain app feedback using QUIS 7.0 international with slight modifications (57, 58). Scores evaluating each item were classified as poor (1–3), intermediate (4–6), and excellent (7–9). The percentage of the responses within each interval was reported as the number of volunteers divided by the total number of volunteers. In order to identify potential patterns, three research team members read the qualitative data (QUIS 7.0 open questions and focus group notes) independently. These data were then converted into codes as the basis of emerging themes (59). The proportion of volunteers suggesting each of the themes that emerged was also estimated. Additionally, to make the app even easier to understand, improvements suggested by the participants (professionals and target population) during this refinement phase were considered and implemented.

Once the feedback was implemented in the final version of the app, a 1-month pilot test with potential users in a real-life environment was performed in order to evaluate the app's relevance, its potential usability, acceptability, credibility, and comprehensibility. The exclusion criteria were the same as those for the abovementioned pilot testing. A total of 39 volunteers were recruited from the previously named healthcare centers by telephone and by the social networks of SAlBi educa, Seville University, and the Health Promotion Area in Sevilla. Volunteers were invited to install the app and use it in their daily lives. After 1 month, the participants completed both the online self-administered sociodemographic and QUIS 7.0 usability questionnaires. Data were analyzed as mentioned above. The percentage of the total number of days on which at least two main eating occasions were tracked was estimated. Additionally, adherence to SAlBi educa was expressed as the percentage of participants who tracked at least 3 days/week with two main eating occasions over time (60).

The methodology used to evaluate feedback and usability of the present app combined both qualitative (focus groups and open questions from the QUIS seven usability questionnaire) and quantitative data (QUIS seven questions rated on a 9-point scale), a methodology frequent in app usability studies (61). Qualitative research sample size determination has always been a contentious issue; our criteria for selecting sample size were based on experience with published studies focusing on app usability testing. In a recent revision focused on usability testing methods in eHealth applications development, including nutrition apps, the mean number of participants for multi-methods was 35.21, with a minimum of 4 participants (61). Additionally, Moumane et al. (57) set the measurements for evaluating mobile applications' usability using ISO standards and the QUIS 7 questionnaire using 32 participants. Our goal was to meet this value as the minimum number of participants. Additionally, we might highlight that in both cases, our sample is representative of the target population and of potential users, which is crucial for qualitative methods (62).

### Statistical Analysis

The data analysis of quantitative data was conducted using the Statistical Package (63). The mean, median, and standard deviation of users' answer punctuation to the QUIS 7.0 questionnaire were included. Significant differences were analyzed using the Mann–Whitney *U* test in Statistica software version 14.0.0.15 (64).

### Ethical Considerations

The Andalusian Regional Government's Biomedical Research Ethics Committee approved the usability study protocol (Ref: 1493-M1-17). In addition, the volunteers signed an informed consent form (Supplementary Figure 1). All personal data obtained in this study were confidential and treated in accordance with the Organic Law Personal Data Protection and Digital Rights Guarantee (65).

## Results

### SAlBi educa Functionalities

The agreed information and requirements resulting from the first three focus groups, involving professionals in healthcare, nutrition, and computer science, were included in the development of SAlBi educa. The general structure of the app's system enables input of the dietary data, nutrient intake to be calculated, and dietary reference values to be compared. It then displays user-tailored advice and food recommendations.

#### Energy Expenditure, Database, and Weekly Meal Planner

**Figure 2:**
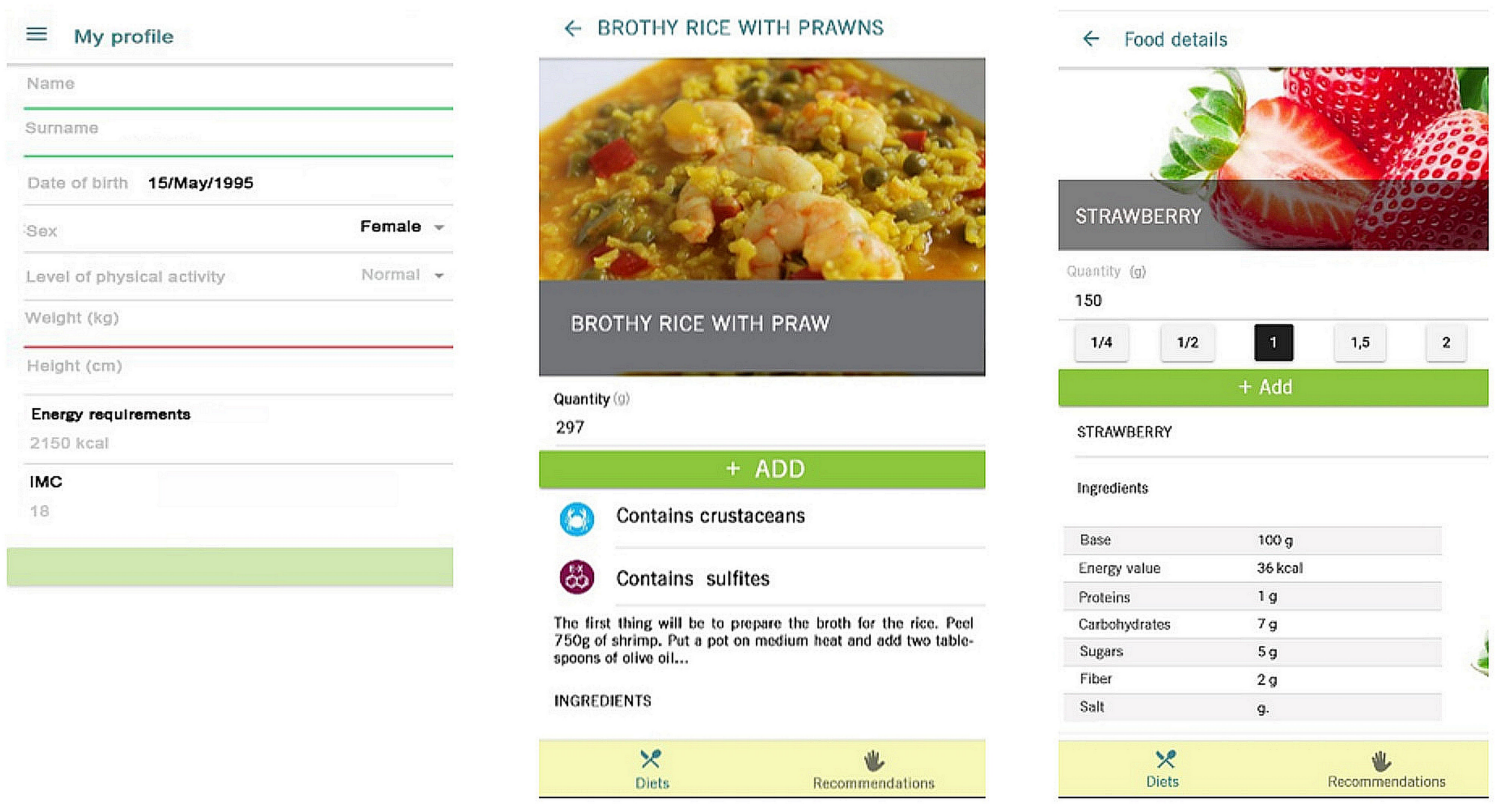
Profile, recipe, and product example.

**Figure 3:**
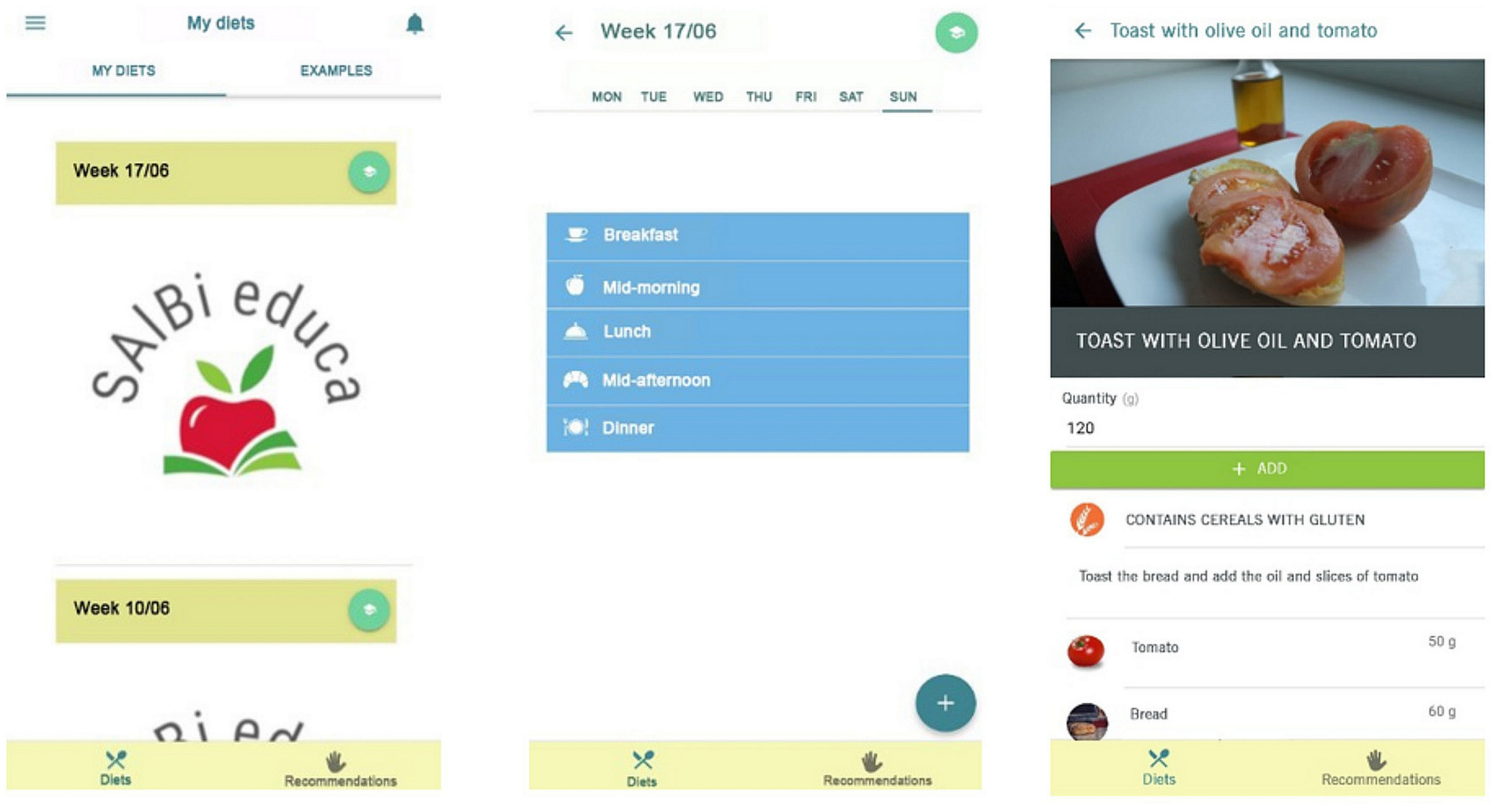
Weekly meal planner and a healthy breakfast recommendation.

Energy expenditure is calculated based on user data (age, sex, weight, and height; Figure 2: left) and level of physical activity according to WHO/EFSA physical activity levels (passive, sedentary, normal, active, and sporty) (63). These data are used to calculate the user's BMI and estimate the total energy expenditure using the Food and Agriculture Organization (FAO)/WHO equations, expressed as kcal (38). The app includes a weekly meal planner to record foods for the five recommended meals in the different days (Figure 3: left and middle). Its database includes more than 300 recipes typical of the Mediterranean diet and more than 1,000 products, both raw and processed food (Figure 2: middle). Each product and dish has its corresponding nutritional information (Figure 2: right) sourced from either the BEDCA database or, in the case of processed products, the label. Additionally, it provides information on allergens and the portion consumed in grams (Figure 2 right and Figure 3 right).

#### Personal Diet and Nutrition Evaluator

**Figure 4:**
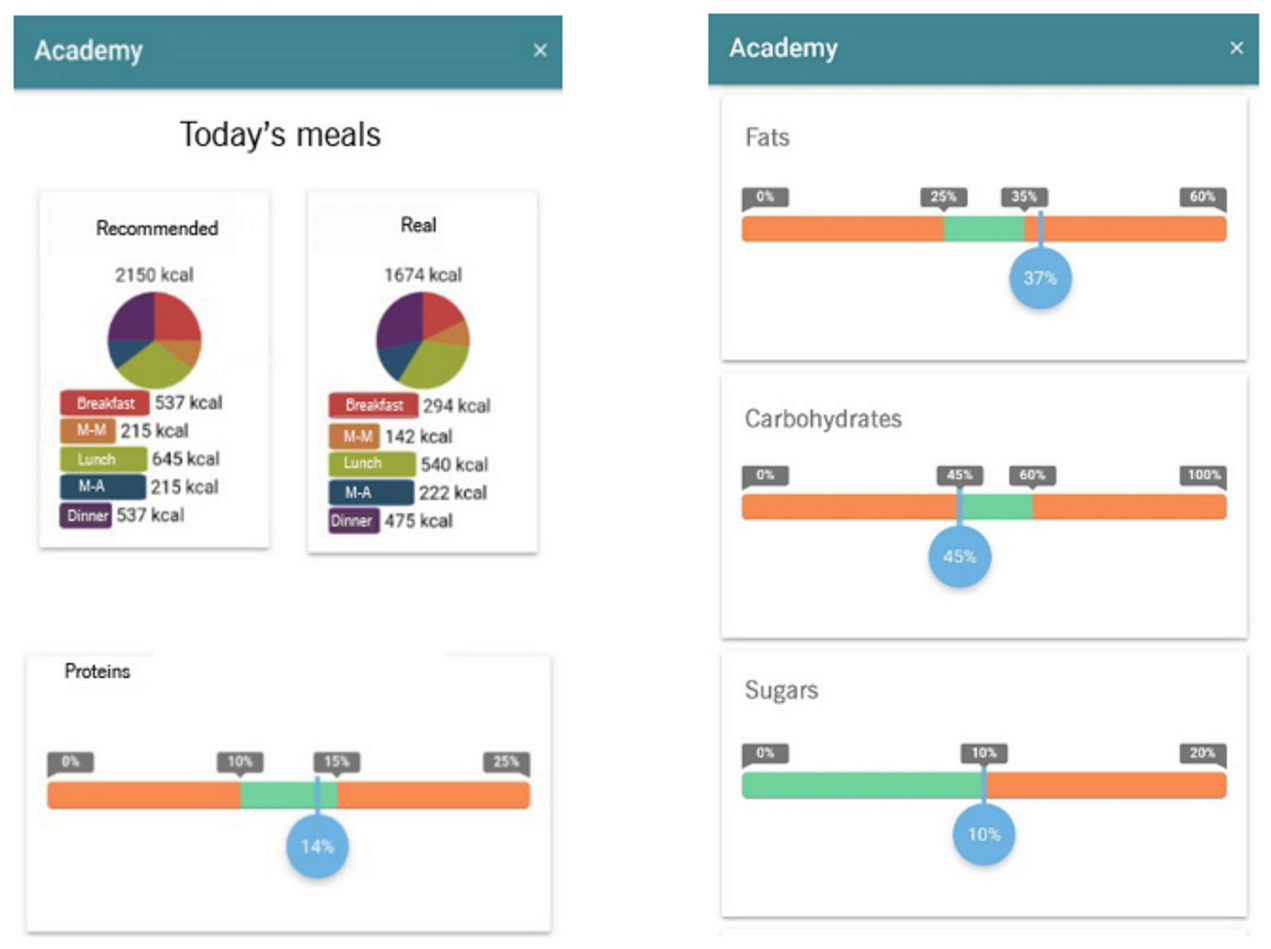
SAlBi educa's self-monitoring tool.

The nutrition self-evaluation tool contains feedback information about the principal nutrients (proteins, fats, carbohydrates, sugar, and fiber), the energy of the recorded diet, and caloric distribution throughout the day (Figure 4). Progress bars use different colors to show whether the diet is within the reference value interval (green) or below or above it (orange).

#### General and Tailored Nutrition Messages

**Figure 5:**
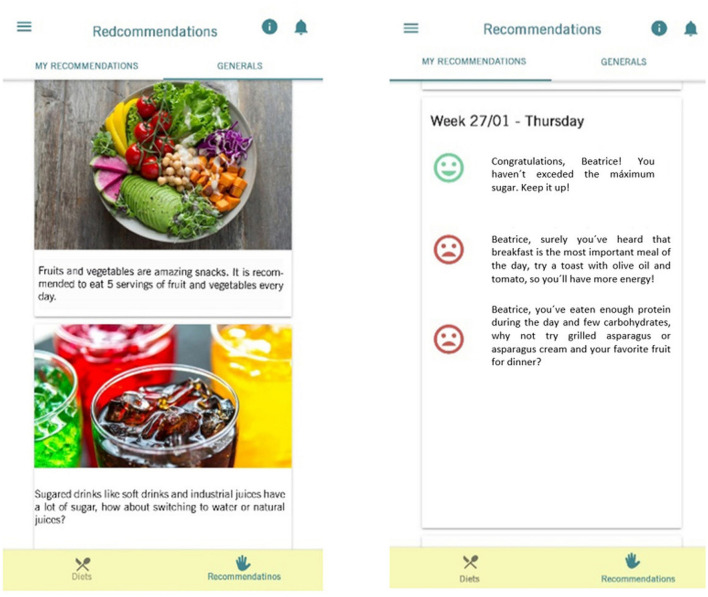
General and tailored messages.

**Figure 6:**
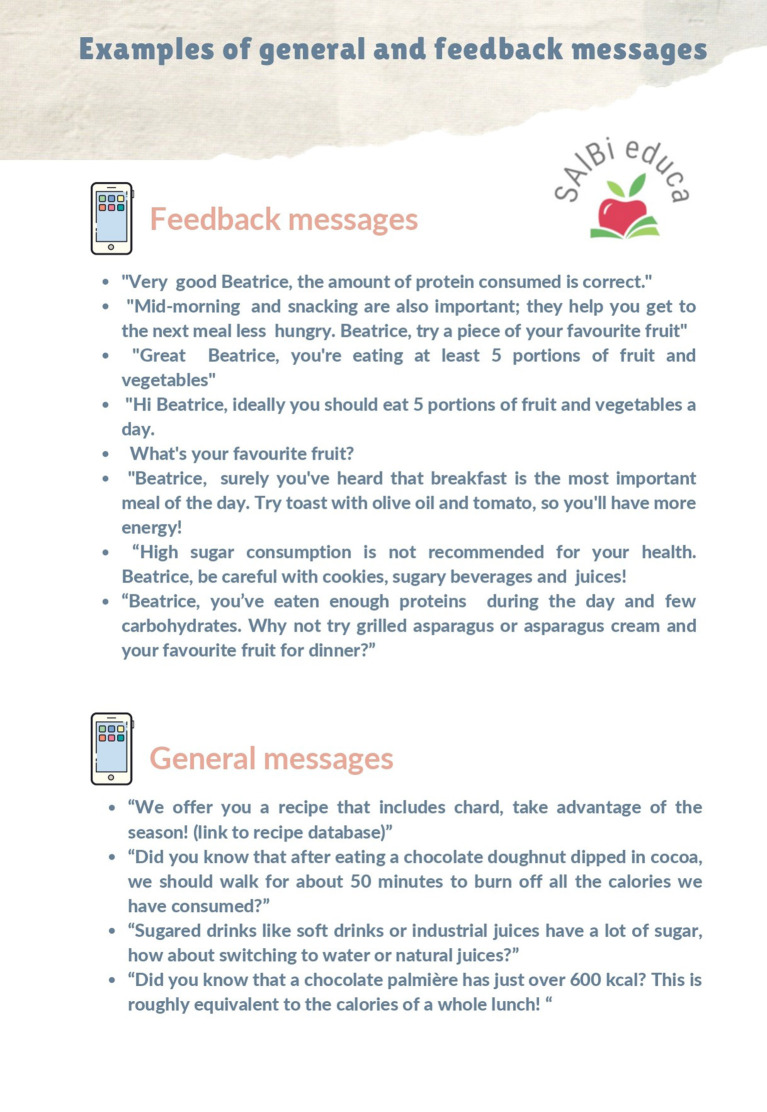
Examples of general and feedback messages.

The objective was first to develop a model message based on the scientific evidence (Table 2). Table 3 summarizes the parameters of the message model used for designing the general and tailored messages developed for SAlBi educa. Totally, 67 feedback and 64 general nutrition messages were designed and developed (Figures 5, 6).

**Table 3:** Characteristics of the message model for its implementation in SAlBi educa.

	**Characteristics**	**References**
**Length**	160 characters	(51, 52, 54, 55)
**Timing**	10.00. User-modifiable	(29)
	16.00–18.00 h	(14, 53)
**Frequency**	1–4 messages/week	(14, 25, 28, 30, 31)
**Content:**		
**General nutrition knowledge**	Healthy diet and nutritional advice	(6, 8, 25, 26, 28–30, 52)
	Examples of everyday situations of increased risk of unhealthy food consumption and healthy suggestions to avoid it	
	Recipes and healthy eating information links	
	Information to promote reflection, discussion, and action	
	Recommendations for healthy snacks and drinks	
	Benefits of fruit and vegetable consumption	
	Benefits of increasing fiber consumption	
	Reducing sugar consumption	
	Information on portion sizes	
	Good food choices	
**Feedback**	Weight progress	(14, 26, 53, 56)
	Macronutrient and energy assessment	
	Motivational, suggesting healthy alternatives, including nutritional advice and simple concepts	
**Tone and language**	Simple	(14, 26, 54)
	Personalized with the user's name	
	Empathetic	

### Professionals and Potential Users' Feedback

A 1-day pilot-test study was conducted to address the multi-perspective approach of users' and healthcare professionals' feedback during the app development phase.

#### Participant Characteristics

A total of 42 volunteers were recruited, including users and healthcare professionals (Table 4). Of the volunteers, 43% had normal weight (all healthcare professionals), 38% were overweight, and 19% were obese (corresponding to users), and 64% were women.

**Table 4:** Characteristics of the study population.

**Characteristics**	**1-day pilot test** **Subtypes (*****n*** **=** **42)**			**1-month usability test** **Subtypes (*****n*** **=** **39)**		
**Age**	**<30**	**30–55**	**>55**	**<30**	**30–55**	**>55**
	2	21	17	12	16	11
**Gender**	**Female**	**Male**		**Female**	**Male**	
	27	15		30	9	
**Education**	**High School**	**Higher**		**High School**	**Higher**	
	13	29		7	32	
**Occupation**	**Professional Healthcare**	**Users**			**Users**	
	23	19			39	
**Socioeconomic level**	**Low**	**Medium**	**High**	**Low**	**Medium**	**High**
	2	37	1	3	33	3
**Marital status**	**Married**	**Single**	**Divorced**	**Married**	**Single**	**Divorced**
	27	11	4	18	16	5
**BMI**	**18.5**–**24.9**	**25**–**29.9**	**>30**	**18.5**–**24.9**	**25**–**29.9**	**>30**
	18	16	8	16	18	5

#### Fulfillment and Feedback Data

Once volunteers had completed the tasks detailed in Figure 1 *via* the app, they immediately received four general and feedback messages. Selected messages were delivered to avoid saturation and user fatigue (Figure 6).

Results of the satisfaction questionnaire (Tables 5, 6) showed that most of the participants (91.9%) declared that their general impression is very good (median and SD of user's answer punctuation are equal to 8 and 1.45, respectively) and pleasant (97.1%). Notably, 91.7% of the participants found it stimulating; 87.1% found it easy to use; and 88.2% agreed that it was flexible (Table 5). All participants considered the screens easy to read and useful, and highlighting the elements on the screen was very useful. Terminology and information are consistent for 100% of the volunteers. Concerning the app learning process, 86.8% of participants thought it easy and 97.2% considered it encouraging to explore it by trial and error; 91.7% of users conducted the tasks with no difficulty (Table 5).

**Table 5:** Results of the satisfaction questionnaire.

**Questions**	**Punctuation [proportion (%)]**					
	**1-day pilot test**			**1-month usability test**		
	**<6**		**>6**	**<6**		**>6**
**How many apps have you used?**	16/39 (41%)		23/39 (59%)	13/39 (33.3%)		26/39 (66.7%)
	**YES**		**NO**	**YES**		**NO**
**Have you used/do you use any nutrition apps?**	3/39 (7.69%)		36/39 (92.3%)	16/39 (41%)		23/39 (59%)
	**YES**		**NO**	**YES**		**NO**
**Do you think nutrition apps can help make changes in eating habits and physical activity?**	41/41 (100%)		0	37/39 (94.9%)		2/39 (5.13%)
	**1–3**	**4–6**	**7–9**	**1–3**	**4–6**	**7–9**
**General user impression of the app**						
Very Bad-Very Good	0	3/37 (8.11%)	34/37 (91.9%)	1/39 (2.56%)	19/39 (48.8%)	19/39 (48.8%)
Frustrating-Nice	0	1/34 (2.94%)	33/34 (97.1%)	2/39 (5.13%)	14/39 (35.9%)	23/39 (59.0%)
Boring-Stimulating	0	3/36 (8.33%)	33/36 (91.7%)	2/39 (5.13%)	24/39 (61.5%)	13/39 (33.3%)
Difficult-Easy	0	4/31 (12.9%)	27/31 (87.1%)	0	10//39 (25.6%)	29//39 (74.4%)
**Screenshots**						
Letters on the app display (Hard to Read-Easy to Read)	0	0	42/42 (100%)	1/39 (2.56%)	8/39 (20.5%)	30/39 (76.9%)
Highlighting of items on the screen (Unhelpful-Very useful)	0	0	40/40 (100%)	0	11/39 (28.2%)	28/39 (71.8%)
Format of the screens was useful (Never-Always)	0	1/41 (2.44%)	40/41 (97.6%)	1/39 (2.56%)	8/39 (20.5%)	30/39 (76.9%)
Screen sequence (Confusing-Clear)	0	0	39/39 (100%)	0	12/39 (30.8%)	27/39 (69.2%)
**Terminology and Information about the app**						
Use of terminology in the app (Inconsistent-Consistent)	0	0	38/38 (100%)	0	7/39 (18%)	32/39 (82%)
Is the terminology appropriate given the task you are doing? (Never-Always)	0	1/38 (2.63%)	37/38 (97.4%)	0	7/39 (18%)	32/39 (82%)
**Learning**						
Learning to use the app (Difficult-Easy)	0	5/38 (13.2%)	33/38 (86.8%)	0	3/39 (7.69%)	36/39 (92.3%)
Exploring the app by trial and error (Discouragement-Feedback)	0	1/36 (2.78%)	35/36 (97.2%)	3/39 (7.7%)	13/39 (33.3%)	23/39 (59.0%)
Tasks can be conducted without difficulty (Never-Always)	0	3/36 (8.33%)	33/36 (91.7%)	2/39 (5.13%)	8/39 (20.5%)	29/39 (74.4%)
Number of steps per task (Too Many-Fair)	0	4/33 (12.1%)	29/33 (87.9%)	5/39 (12.82%)	9/39 (23.1%)	25/39 (64.1%)
Steps to complete a task follow a logical sequence (Never-Always)	0	3/39 (7.69%)	36/39 (92.3%)	0	12/39 (30.8%)	27/39 (69.2%)
**System capacity**						
System speed (Too Slow-Very Fast)	0	1/39 (2.56%)	38/39 (97.4%)	1/39 (2.56%)	10/39 (25.6%)	28/39 (71.8%)
The system is reliable (Never-Always)	0	0	40/40 (100%)	1/39 (2.56%)	6/39 (15.4%)	32/39 (82.1%)
Correct my mistakes (Difficult-Easy)	0	2/36 (5.56%)	34/36 (94.4%)	2/39 (5.13%)	11/39 (28.2%)	26/39 (66.7%)
System ease of use depends on my level of experience (Never-Always)	0	1/40 (2.5%)	39/40 (97.5%)	4/39 (10.26%)	18/39 (46.2%)	17/39 (43.6%)
**Multimedia**						
Quality of illustrations and photographs (Bad-Good)	0	0	39/39 (100%)	2/39 (5.13%)	11/39 (28.2%)	26/39 (66.7%)
Colors used (Artificial- Natural)	0	0	39/39 (100%)	1/39 (2.56%)	7/39 (18%)	31/39 (79.5%)
**Nutritional messages**						
Clear messages (Disagree - Agree)	0	3 (7.69%)	36/39 (92.3%)	6/39 (15.38%)	10/39 (25.6%)	23/39 (59%)
Useful messages (Disagree - Agree)	0	0	39/39 (100%)	5/39 (12.82%)	11/39 (28.2%)	23/39 (59.0%)
Relevant messages (Disagree-OK)	0	1/38 (2.63%)	37/38 (97.4%)	4/39 (10.26%)	14/39 (35.9%)	21/39 (53.8%)
Persuasive potential to improve diet/physical activity (Very Low Potential - High Potential)	0	5/36 (13.9%)	31/36 (86.1%)	6/39 (15.38%)	14/39 (35.9%)	19/39 (48.7%)
**What part of the design of SAlBi educa do you consider to be the most motivating/useful? (Proportion)**	• Images (4/42) • Tailored feedback messages (29/42) • Clarity of messages (28/42) • Graphics (30/42) • Recipe examples (4/42)			• General recommendations (22/39) • Color and quality of photos (2/39) • Graphics (23/39) • Feedback messages (23/39) • Recipes (1/39)		
**If you could make any modifications in SAlBi educa to improve it, what would you change? (Proportion)**	• Database with most common dishes (20/42) • Alphabetical order of dishes and products (12/42) • Starting tutorial (15/42) • More intuitive tool for removing a dish or product (24/42) • Copy meals functionality (25/42)			• Improve food search (9/39) • Healthy alternatives (5/39) • Possibility of introducing food and dishes and type of cooking (14/39)		
	**YES**		**NO**	**YES**		**NO**
**Would you use SAlBi educa in the future?**	38/40 (95%)		2/40 (5%)	29/39 (74.4%)		10/39 (25.6%)
**Would you recommend SAlBi educa to other users?**	38/38 (100%)		0	30/39 (76.9%)		9/39 (23.1%)
**Is there another nutrition app you would prefer to use instead of SAlBi educa?**	1/32 (3.13%)		31/32 (96.87%)	4/39 10.3%)		35/39 (89.7%)

**Table 6:** Results of satisfaction users' punctuation to the QUIS 7.0 questionnaire.

**Satisfaction measures**	**One-day pilot test**			**1-month usability test**		
	**Mean**	**Median**	**SD**	**Mean**	**Median**	**SD**
General impression	7.43	8	1.45	6.56	7	1.59
Screenshots	7.84	8	1.65	7.25	7	1.56
Terminology & information	7.80	8	1.64	7.29	7	1.39
Learning	7.28	8	2.13	7.12	7	1.72
System capacity	7.84	8	1.68	6.85	7	1.75
Multimedia	8.45	9	2.29	7.14	7	1.73
Nutritional messages	7.31	7	2.57	6.42	7	2.25

Notably, 92.3% of volunteers stated that both the general and tailored nutrition messages are clear, useful (100%), and relevant (97.4%), while 86.1% agreed that they have the potential to persuade people to improve their diet and physical activity. Users gave a median value of 7 (SD 2.57) for SAlBi educa's feedback and general messages. A total of 95.0% of participants would use SAlBi educa in the future and recommend it to other users (Table 5). Users considered the most useful and motivating elements to be the tailored feedback messages, nutrient assessment charts (graphics), and message clarity (Table 5). Although in minor proportion, participants considered images and recipe examples as motivating tools. Fulfillment data demonstrate that SAlBi educa satisfies the interest and expectation for a nutrition app among both health professionals and the target population.

**Figure 7:**
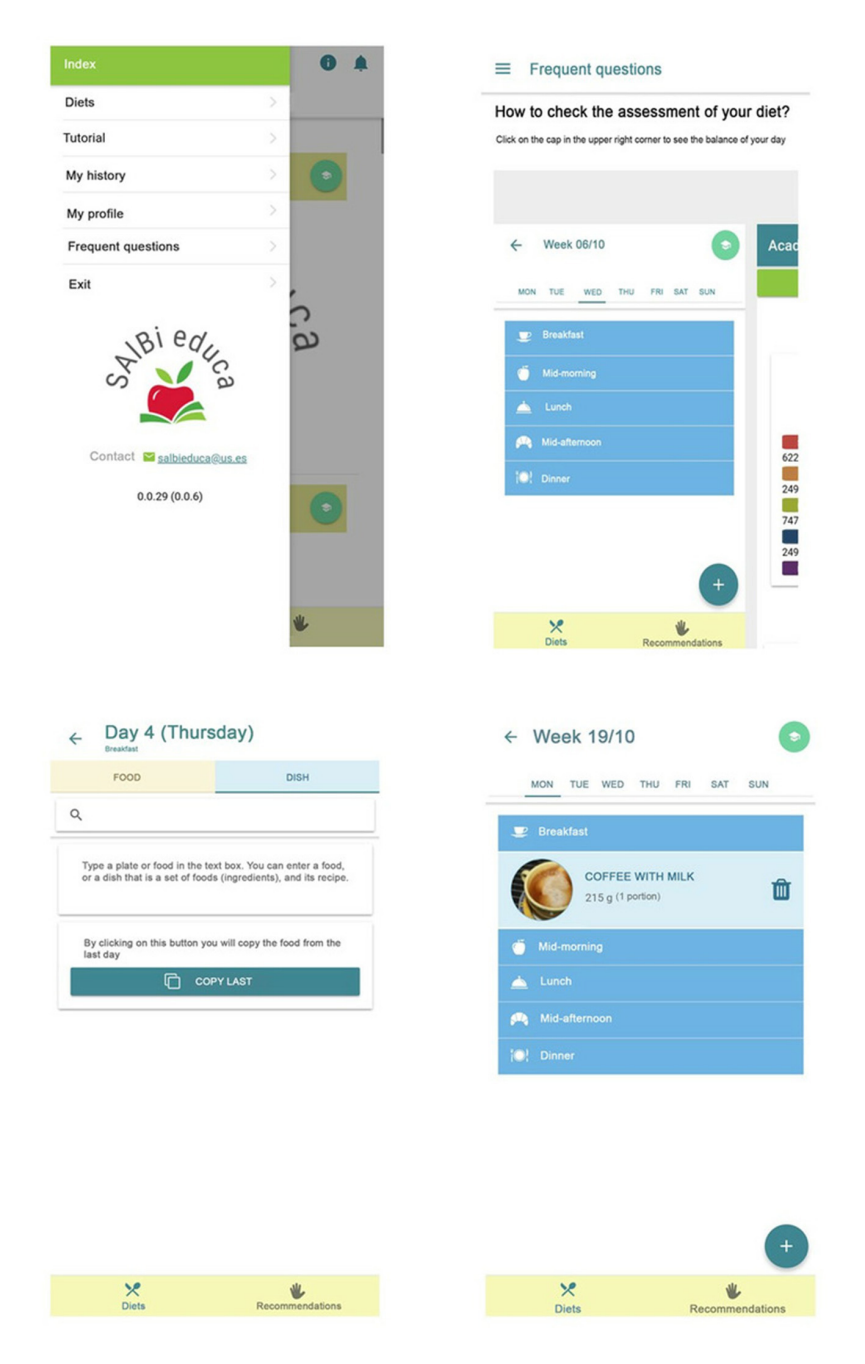
Improvements implemented in SAlBi educa last version.

Participants also proposed improvements as follows: i) more traditional Mediterranean dishes in the database and displaying them in alphabetical order; ii) a more intuitive way of removing a dish or product and changing their quantities; iii) adding a starting tutorial on their use; and iv) the possibility of copying meals from previous days (Table 5). It should be noted that all the volunteers' suggestions have been implemented in the latest version of the app (Figure 7).

### Usability Evaluation of SAlBi educa

Once the latest version of the app incorporating the contributions from professionals and the target population was ready, a 1-month pilot test for usability testing was performed, involving potential users in a real-life environment. A total of 39 volunteers were recruited, of which 77% were women (Table 4). This was not intentional and we found that women were more motivated to take part, as usual in these kinds of studies (female participants ranged between 60 and 88.5%) (4, 66–69).

After a month of using the app, volunteers thought that SAlBi educa is nice (59%) and easy to use (74%) (Table 5). The general impression was also very good, scoring the median value of 7 (SD 1.59). More than 75% of the volunteers believed that the screens were very useful. Users highlighted that the terminology was very consistent and appropriate (82%). Of the volunteers, 92% underlined that SAlBi educa was easy to learn. More than 84% of the volunteers declared that nutritional messages were clear and useful, the median value being 7 (SD 2.25; Table 6). More than 70% would use and recommend SAlBi educa in the future. Statistical analysis showed significant differences within educational level for individual questions regarding “Nutritional messages” (Table 7) only. The highest scores were given by people who were high school graduates. Notably, no significant differences were found for “Terminology & Information” questions between participants at different levels of education. This means that the individual participant's ability to understand the app is not dependent upon his/her level of education. Furthermore, no significant differences were found within gender (Table 7).

**Table 7:** Satisfaction punctuation (median) at different educational levels and gender.

**Satisfaction measures**	**1-month usability test**			
	**Educational level**		**Gender**	
	**High School**	**Higher**	**Male**	**Female**
GI	6	7	7	7
SC	7	8	8	7
TI	7	8	8	7
LE	7	8	8	7
SY	7	7	7	7
MU	7	7.5	7	8
NU	6^a^	7.5^b^	7	7
Total satisfaction	7	7	7	7

*GI, general impression; SC, screenshots; TI, terminology and information; LE, learning; SY, system capacity; MU, multimedia; NU, nutritional messages*;

ab *means significant differences within educational level for nutritional messages questions, p < 0.05*.

Additionally, volunteers stated that the general and tailored feedback messages, as well as the self-monitoring (graphics), were the most motivating and useful parts of SAlBi educa. Participants tracked 62.5% of total days with at least two main eating occasions, which means that participants tracked a mean value of 4.4 days/week (considering 7 days at 100%). The mean number of meals entered per day was 3.98 (± 0.61). At week 4 (the end of the study), adherence was still at 81.3% of participants who were tracking at least 3 days/week with two main eating occasions (87.5% for week 2 and 78.1% for week 3). These data show a good acceptance of the app over at least 1 month.

## Discussion

Developments in new technologies have enabled developers to create innovative tools, such as nutrition apps, to tackle health promotion and healthy eating habits (14, 70). Nutritional education through apps with tailored messages and notifications is a powerful tool since they provide individualized feedback and self-motivation, mimicking the person-to-person dietary counseling process.

This study includes the design and development of the SAlBi educa nutrition app by both professionals and the target population, as well as the usability data of potential users. In summary, in just one single app, SAlbi educa offers users unique tools such as tailored feedback messages, evaluating graphics, and traditional Mediterranean recipe examples, while many apps lack local food composition database support (69, 71). Users find SAlbi educa stimulating, easy to use, and useful for persuading people to improve their diet. Yet, as far as we know, none of the apps reported in the literature (14–17) include sugar intake evaluation, caloric distribution throughout the day, or suggest examples of dishes or foods to balance the user's diet all in the same app.

Message length is not always described in the studies. However, when it is mentioned (4/15 studies), all of them agree on 160 characters (about 2–3 sentences) (30, 52, 54). In most cases, message frequency was a key aspect in maintaining user adherence and reducing app use abandonment (14). Therefore, the authors agree that the number of outreach messages sent should be gradually increased in order to maintain expectations, as well as alternating the type of message to make them more varied and interesting (29). Five of the fifteen studies declared the frequency used as being between 1 and 4 messages/week (Table 2).

Some authors displayed the message according to a predetermined schedule to avoid interfering with users' daily chores (14). However, others stressed that it would be important to adjust the messages to the users' routine, making the massaging schedule user-specific (53).

Furthermore, the authors agreed that personalization was one of the keys to an application's success (72). Those messages that were individualized and personalized had a greater impact on users than those that were general for the entire population. Customization included the name of each user and a design that adapted to what each of them recorded. Others found that messages with an empathetic tone were much more effective and persuasive in improving diet (14, 54).

A total of eleven of the fifteen studies included text messages with general nutritional content (6, 8, 25, 28–30, 50–52, 54, 55). However, only four of the sixteen studies included tailored feedback messages (14, 26, 31, 53). This fact highlights the originality of SAlBi educa.

Previous research on smartphone apps for changing behavior has highlighted that users find receiving graphic commentaries on success by monitoring and tracking features both positive and valuable (52, 55, 69, 71–73). Specifically, users of the Well-D app reported that the most liked feature was the diet monitoring and the real-time feedback about nutrient and food intake (74). In fact, behavioral science research has established that self-monitoring and receiving feedback on performance are key techniques for changing behavior. In other words, they are effective in reinforcing behavioral changes (14). The results in this study agree, since volunteers highlighted the tailored feedback messages and self-evaluating graphics as among the most motivating tools.

Recent studies reveal that apps that include self-monitoring and tailored feedback are more effective in achieving significant reductions in body weight, the waist–hip ratio, and BMI, while also increasing user knowledge concerning nutrition and healthy diet. This is because, since such feedback commands greater attention, it is more intensively processed and is better appreciated by the population (9, 15, 17, 18, 54). Further research will, therefore, be conducted to demonstrate the efficacy of the SAlBi educa app on the abovementioned parameters.

Primary healthcare professionals are in a unique position to provide effective dietary counseling. Communication between these professionals and citizens is one of the principal sources of information and training when tackling nutrition and physical activity. Maderuelo-Fernandez et al. demonstrated that primary care dietary counseling in five different countries (Holland, Italy, Spain, UK, and USA) improved eating habits (75). Despite all efforts, however, overweight and obesity continue to rise worldwide (76).

New technologies provide us with an opportunity to complement the face-to-face nutrition counseling given to citizens. Healthcare professionals opine that implementing the new technologies in primary care nutrition counseling would be useful for the following reasons: i) to improve the user's eating habits; ii) to empower patients; iii) to increase patient motivation due to the self-evaluation provided continuously and almost ubiquitously with a great level of privacy; and iv) to enable a logical connection between dietary modification and goals achieved to be established, thus, increasing patients' sense of self-control and self-efficiency (4).

**Figure 8:**
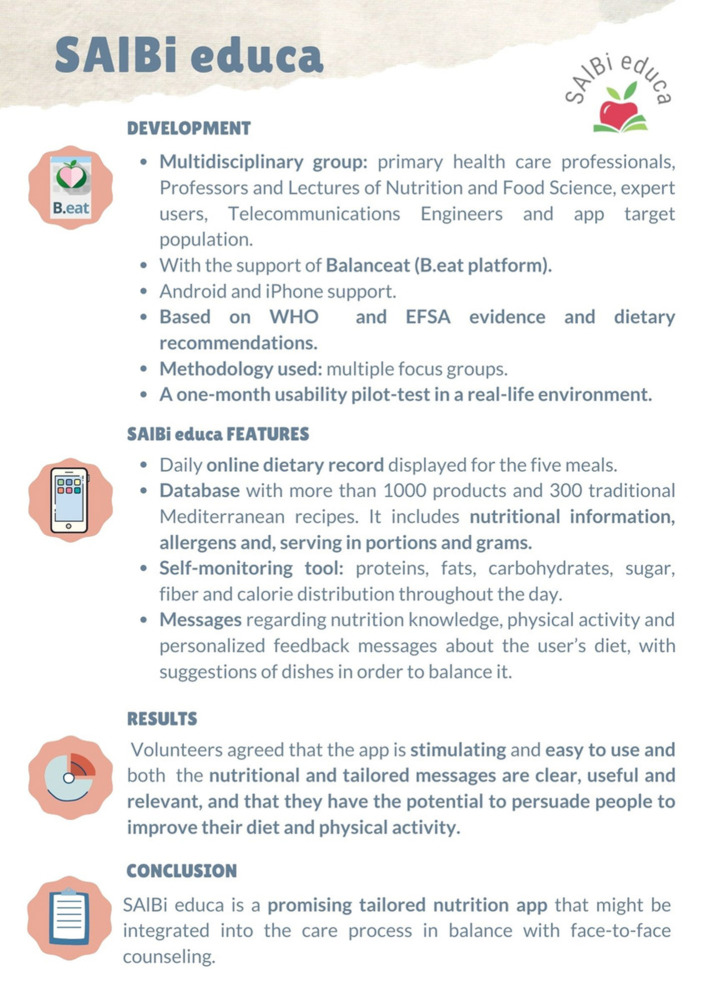
Synthesis of SAlBi educa.

SAlBi educa aims to be a nutritional education tool implemented in primary care nutrition counseling. The use of the app does not aim to substitute for the healthcare practitioner. On the contrary, it might be seen as an additional supporting tool that permits the practitioner to better monitor diet adherence, thus saving time. Additionally, the practitioner can provide personalized advice, improving service quality. In agreement with other authors, SAlBi educa aspires to be integrated into the healthcare process in tandem with face-to-face counseling (4). Further pilot interventions in a real environment will be needed to demonstrate its efficacy. Figure 8 provides a synthesis of SAlBi educa's development, features, and the results and conclusion of this study for developing a clinical practice guide. Once SAlBi educa has demonstrated its effectiveness, the Andalusian Regional Public Health Service (SAS) has expressed its intention to integrate the app into the user's electronic medical record. Due to the current situation created by the COVID-19 pandemic, not only does this type of nutrition app gain importance as a complement, but also sometimes as an alternative to primary care nutrition counseling, when health professionals find themselves obliged to direct their efforts toward other priorities.

Several limitations must be considered when interpreting the results of this present study. Several types of user data entered into SAlBi educa, such as weight, height, and physical activity, are self-declared. Therefore, an underestimation of the general energy expenditure and BMI calculated by the app might occur. The role of health professionals in providing authentic anthropometric measurements is crucial. Although the database is quite large (>1,000 products and >300 recipes) and based on the typical Mediterranean diet, there is a risk that certain food products or recipes might not be included in the database. However, users will always be able to send their suggestions for inclusion in the app database. Additionally, SAlBi educa is set up in Spanish. Therefore, at the moment, it is aimed at Spanish speakers. However, the basic methodology could be extended to other cultures, by means of including typical dishes in its database, and languages. This might be considered for including in further updates. As yet, the effectiveness of SAlBi educa in terms of improving eating habits has not been assessed; further research will focus on a control trial intervention study to demonstrate this effectiveness.

## Conclusion

SAlBi educa, the app developed, is an innovative nutritional education tool. It includes a weekly dietary record, nutrients and energy self-monitoring graphics, general and tailored feedback messages, and examples of traditional Mediterranean recipes. It has the potential to become an effective solution for supporting nutrition counseling in primary healthcare. The usability study conducted in a real-life environment shows that SAlBi educa is clear, useful, stimulating, and easy to use, and might have the potential to persuade people to improve their diet and physical activity. SAlBi educa has been demonstrated to be understandable independently of the educational level and more appreciated by people with a lower level of education.

## Summary

SAlBi educa, a tailored nutrition app, has been developed to promote healthy eating habits through the Mediterranean diet, currently a reference for a healthy diet. This study describes its design and development process, based on scientific evidence and nutritional recommendations from international organizations and conducted by professionals. Additionally, during the development of the app, feedback from users and health professionals was implemented. Finally, the usability data of the target population was evaluated. The main features include nutrients and energy self-monitoring graphics, general and tailored feedback messages, and examples of traditional Mediterranean recipes to improve the user's diet. Length, frequency, timing, content, tone, and language of feedback messages were selected and based on the scientific literature. Users declare that SAlBi educa is easy to use and easy to learn, as well as motivating for improving healthy eating habits. Nutrition education is a big issue in primary healthcare due to its potential to tackle the development of chronic diseases. This already-tested app would help to support nutrition counseling, not only for professionals in their mission of health promotion but also to principally enable the general adult population to make informed decisions and improve their diet.

## Data Availability Statement

The original contributions presented in the study are included in the article/Supplementary Material, further inquiries can be directed to the corresponding author/s.

## Ethics Statement

The studies involving human participants were reviewed and approved by Andalusian Regional Government's Biomedical Research Ethics Committee. The patients/participants provided their written informed consent to participate in this study.

## Author Contributions

MG-R, AT, MG-P, and AC: conceptualization and writing—original draft preparation. AC-L, MG-R, AT, MG-P, and AC: methodology and data curation. MG-R, AC-L, ML-N, ES-G, MT-B, MS-B, MS-C, MB-V, FP-B, AT, MG-P, and AC: formal analysis and writing—review and editing. AT, MG-P, and AC: supervision. All authors have read and agreed to the published version of the manuscript.

## Funding

This study was funded by the Andalusian Regional Government of Health and Family (Consejería de Salud y Familia, Junta de Andalucía) (Innovative Project on Health; grant number PIN-0050-2018), and Balanceat Advanced Nutrition Technologies S. L.

## Conflict of Interest

The authors declare that this study received funding from Balanceat Advanced Nutrition Technologies S.L. The funder had the following involvement in the study: Technological development of the app. The funder was not involved in the study design, collection, analysis, interpretation of data, the writing of this article or the decision to submit it for publication.

## Publisher's Note

All claims expressed in this article are solely those of the authors and do not necessarily represent those of their affiliated organizations, or those of the publisher, the editors and the reviewers. Any product that may be evaluated in this article, or claim that may be made by its manufacturer, is not guaranteed or endorsed by the publisher.
