# Supplementary material for: SAlBi educa (Tailored Nutrition App for Improving Dietary Habits): Initial Evaluation of Usability
Source: Front Nutr. 2022 Apr 19;9:782430. doi: 10.3389/fnut.2022.782430 (PMC9063930; doi:10.3389/fnut.2022.782430)
Supplement: Supplementary file 1 [file Image_1.pdf]

Supplementary Material

INFORMED CONSENT - PATIENT INFORMATION

Before signing this informed consent form, please read the following information carefully and ask any questions you may have.

Nature:

You will be given questionnaires on sociodemographic, anthropometric and satisfaction data. This is in order to evaluate the usability of the nutrition application that is being developed in the research project entitled "IMPROVING THE EATING HABITS OF THE ATTENDANTS TO THE DIETARY ADVICE IN PRIMARY CARE THROUGH THE USE OF A MOBILE NUTRITION APPLICATION". The project aims to evaluate the effectiveness of new technological strategies (nutrition app) on improving eating habits and healthy lifestyles in adults attending the Dietetic Advice sessions in Primary Care.

Significance:

This study responds to the need to support nutritional intervention programmes. It relies on public and private entities, involves both the largest number of people possible, involving multidisciplinary teams promoting permanent changes in the subjects' routines, focusing principally on improving eating habits and healthy lifestyles. To this end, the efficacy of new technological intervention strategies will be developed and evaluated, such as nutritional education through a nutrition app, implemented in the Dietary Counselling carried out in Primary Care. The aim is to increase adherence to a balanced diet and healthy lifestyles in adults attending the Dietary Counselling.
Your participation in the study can help you to improve your eating habits and lifestyle, as well as to increase your knowledge of balanced eating and healthy lifestyles, as you will have access to dietary advice through an innovative tool, the nutrition app. It is also possible that you will not get any direct benefit from participating in the study. However, it is likely that some of the information obtained may benefit others in the future and may contribute to a better understanding of the effect of new technologies, in other words nutrition apps, on improving eating habits.

Implications for the patient:

- Participation is entirely voluntary.
- The patient may withdraw from the study at any time, without giving any explanation and without this having any repercussions on their medical care.
- All personal data obtained in this study are confidential and will be treated in accordance to the Organic Law on Personal Data Protection 3/2018.
- The information obtained will be used exclusively for the specific purposes of this study.

Risks of the research for the donor/patient:

The study does not involve any risk to your health as you will not be required to undergo any analytical tests or take any drugs.

If you require additional information, you can contact the researcher responsible for the project (Dr. A. B. Cerezo López) by telephone: 954 556 760 or by e-mail: acerezo@us.es

INFORMED CONSENT - WRITTEN PATIENT CONSENT

IMPROVING THE DIETARY HABITS OF PRIMARY CARE DIETETIC COUNSELLING ATTENDEES THROUGH THE USE OF A MOBILE NUTRITION APPLICATION

I (Name and Surname): ....
DECLARE THAT:

- I have read the information document accompanying this consent (Patient Information).

- I have been able to ask questions about the study IMPROVING THE EATING HABITS OF PRIMARY CARE DIETARY ADVICE ATTENDEES THROUGH THE USE OF A MOBILE NUTRITION APP

- I have received sufficient information about the study IMPROVING THE EATING HABITS OF PRIMARY CARE DIETARY ADVICE ATTENDEES THROUGH THE USE OF A MOBILE NUTRITION APPLICATION. I have spoken to the researcher responsible for the project: ....

- I understand that my participation is voluntary, and that I am free to participate or not in the study.

- I have been informed that all data obtained in this study will be confidential and will be treated in accordance with the Organic Law on Personal Data Protection 3/2018.

- I have been informed that the information obtained will only be used for the specific purposes of the study.

I understand that I can withdraw from the study:

- Whenever I want
- Without having to give explanations
- Without detriment to my medical care.

I freely agree to participate in the project entitled IMPROVING THE EATING HABITS OF PRIMARY CARE DIETARY ADVICE ATTENDEES THROUGH THE USE OF A MOBILE NUTRITION APPLICATION.

Patient's, or, where applicable his/her legal representative's signature:

Project reporting researcher's signature:

Supplementary Figure 1. Informed consent form. It was translated to English for purposes of clarity
